# A case study of 2019-nCOV cases in Argentina with the real data based on daily cases from March 03, 2020 to March 29, 2021 using classical and fractional derivatives

**DOI:** 10.1186/s13662-021-03499-2

**Published:** 2021-07-20

**Authors:** Pushpendra Kumar, Vedat Suat Erturk, Marina Murillo-Arcila, Ramashis Banerjee, A. Manickam

**Affiliations:** 1 Department of Mathematics and Statistics, School of Basic and Applied Sciences, Central University of Punjab, Bathinda, Punjab 151001 India; 2 Department of Mathematics, Faculty of Arts and Sciences, Ondokuz Mayis University, Atakum, 55200 Samsun, Turkey; 3 Instituto Universitario de Matematica Pura y Aplicada, Universitat Politècnica de València, 46022 Valencia, Spain; 4 Department of Electrical Engineering, National Institute of Technology, Silchar, India; 5 School of Advanced Sciences & Languages, Department of Mathematics, VIT Bhopal University, Kottri Kalan (Village), 466 114 Sehore (District), Madhya Pradesh India

**Keywords:** 34D20, 26A33, 47H10, 92D30, COVID-19, Argentina, Mathematical models, TRR algorithm, Atangana–Baleanu non-classical derivative

## Abstract

In this study, our aim is to explore the dynamics of COVID-19 or 2019-nCOV in Argentina considering the parameter values based on the real data of this virus from *March* 03, 2020 to *March* 29, 2021 which is a data range of more than one complete year. We propose a Atangana–Baleanu type fractional-order model and simulate it by using predictor–corrector (P-C) method. First we introduce the biological nature of this virus in theoretical way and then formulate a mathematical model to define its dynamics. We use a well-known effective optimization scheme based on the renowned trust-region-reflective (TRR) method to perform the model calibration. We have plotted the real cases of COVID-19 and compared our integer-order model with the simulated data along with the calculation of basic reproductive number. Concerning fractional-order simulations, first we prove the existence and uniqueness of solution and then write the solution along with the stability of the given P-C method. A number of graphs at various fractional-order values are simulated to predict the future dynamics of the virus in Argentina which is the main contribution of this paper.

## Introduction

The coronavirus disease 2019 (COVID-19) is considered as the most dangerous epidemic disease of this decade, which appeared for the first time in Wuhan (China) in the last month of 2019. It has been observed that coronavirus disease 2019 (COVID-19) is a vital health concern as it can be fatal specially in old-aged people. SARS-CoV-2 virus causes COVID-19 disease. Researchers and medical practitioners have much information about the death due to the clinical disease but they do not know much about its pathobiology. It has been seen that the characteristics of cellular responses to COVID-19 are not clearly known and understood, but based on the previous studies on SARS-CoV, a predictable sequence of events can be hypothesized. According to the cells that SARS-CoV infects, COVID-19 can be separated into three periods that coincide with the clinical parts of the disease [1].

The structure of SARS-CoV-2 needs to be understood. It has been found that SARS-CoV-2 is part of the family of beta-coronavirus, some of which caused two other epidemics named as MERS-CoV and SARS-CoV as can be found in Ref. [2]. In [3] Hui et al. analyzed the structure of such coronaviruses. According to the World Health Organisation (WHO), the rate of death due to SARS-CoV-2 is lower than the one due to SARS-CoV though the rate of transmission is higher in the case of SARS-CoV-2 than SARS-CoV. The RNA structure of different coronaviruses has been stated in [4]. It has been observed that SARS-CoV-2 has distinctive spike proteins and presents a specific peptide, namely PrrA, which allows one to divide the spike protein using cellular protease enzymes in order to disseminate the virus from the cell of the host easily [5, 6]. So, the rate of transmission of SARS CoV-2 is much higher and this is the prime cause of its spreading throughout the globe in a very short period of time.

It has been observed that as soon as SARS-CoV-2 makes its entrance into the epithelial cell of alveoli of the respiratory tract of human being, the virus activates the immune response of human being due to the fast multiplication rate of SARS-CoV-2 cells. After that, the pulmonary tissue from the respiratory tract of human being is damaged causing the stimulation of more and more white blood cells. This process is known as the cytokine release syndrome [7], which can cause a multiple organ failure [8].

There are a number of stages included in the infection of COVID-19. The initial stage is asymptomatic and covers 1 to 2 days after getting infected. It has been noticed that after inhaling the virus it attaches to epithelial cells from the nasal cavity of human being and the virus starts the process of replication [9]. In this initial stage, infected people are able to spread the infection. During the second stage the virus is able to spread and move quickly down to the respiratory tract along with the conducting passage of air [10]. It has been observed that for 80% of the infected individuals the COVID-19 illness will be lenient and it is restricted to upper as well as conducting passages of air. The third stage of the disease involves hypoxia and ground glass infiltrates along with the growth of acute respiratory distress syndrome (ARDS). It has been seen that 20% of the infected individuals will move to third stage of the disease and they will develop pulmonary invasions which will lead to severe disease. From the initial estimation the rate of severity is about 2% but it varies with age [1]. In this stage SARS-Cov-2 virus is able to reach the units of the lung which are responsible for exchange of gas and infect the type II cells of alveoli. SARS-CoV is able to spread within type II cells and huge viral particles are liberated [11]. Old-aged people are at high risk since they have a low immune response, which can lead to severe consequences [12].

Mathematical models are the best way to describe the dynamics of the disease. Many mathematical models have been provided to illustrate the behavior of COVID-19 by using integer- and fractional-order derivatives [13–18]. A fractional-order COVID-19 model with delay has been proposed in [19]. *Atangana et al.* in [20] have discussed a mathematical model of COVID-19 with deterministic and stochastic approaches. The authors in [21–23] have also simulated the dynamics of 2019-nCOV by using effective mathematical models. A study of a fractional-order model of HIV is given in [24]. The authors in [25] proposed a research on the dynamics of a population model. In [26], a COVID-19 model along with necessary awareness programs has been explored. The roles of isolation and quarantine measures for COVID-19 outbreaks are given in [27]. The authors in [28] have solved a model of huanglongbing transmission with an effective numerical algorithm. A psychological model in fractional-order sense has been introduced in [29]. *Kumar et al.* in [30] have described a time-delay fractional-order mathematical model of oncolytic virotherapy. Many other epidemics have been described by using fractional mathematical models [31, 32]. Recently, the authors in [33] studied the structure of rabies, and canine distemper virus epidemics by using generalised Caputo non-classical derivative. Two fractional-order mathematical models for describing mosaic disease have been given in [34]. A environmental study in the fractional-order sense is given in [35].

This paper is formulated to trace the dynamics of COVID-19 cases in Argentina by using Atangana–Baleanu type fractional mathematical model. The study is organized as follows: In Sect. 2, we describe the integer-order model dynamics. In Sect. 3, we plot the real-data cases of COVID-19 in Argentina and calculate the parameter values for our simulations. Section 4 is organized in a number of sub-sections where we simulate the fractional-order model and perform all theoretical and graphical simulations. Finally in Sect. 5, we present our conclusions about the study.

## Model description

In this paper, we consider a compartmental mathematical model [36]. Natural births and death rates have not been considered in the model as those have no impact on the short-term outbreaks of COVID-19. The mentioned model focuses on two distinct groups of susceptible individuals: susceptible individuals given by $S(t)$ and confined individuals who follow lockdown or confinement intervention partially as the confinement is not perfect. We symbolize the class of confined individuals as $C(t)$. The rest of the population is compartmentalized as follows: exposed individuals $E(t)$ at time *t*, asymptomatic (having no clinical symptoms or mild symptoms) individuals $A(t)$, quarantined symptomatic infectious individuals $Q(t)$, hospitalized or isolated individuals $H(t)$ and recovered individuals $R(t)$.

In the given model, *p* is the transmission rate from confined susceptible humans to rejoined unconfined susceptible humans, *qγ* is the exposure rate of the asymptomatic humans, $r_{4}\sigma _{2}$ is the progression rate from severely infected to hospitalization and quarantine rate is given by $(1-q)\gamma $. $r_{1}\sigma _{1}$ denotes the transmission rate of the asymptomatic humans to become severely infectious. $r_{2}\sigma _{1}$ shows the transmission rate of confinement or hospitalization to isolation of the asymptomatic group. The natural recovery of soft symptomatic humans is represented by the rate $(1-r_{1}-r_{2})\sigma _{1}$, whereas $(1-r_{3}-r_{4})\sigma _{2}$ is the recovery rate for *Q* classes. In the model, $\delta _{h}$, $\delta _{q}$, and $\delta _{a}$ are stood for the COVID-19 death rates in the given model classes, respectively. The structure of the non-linear model based on the given parameters explanation is provided as follows:

1 $$ \textstyle\begin{cases} \frac{dS}{dt}(t) =pC(t)-cS(t)- [ \frac{\beta ( A(t)+\eta Q(t) ) }{N(t)} ]S(t), \\ \frac{dC}{dt}(t) =cS(t)-pC(t)-(1-\epsilon ) [ \frac{\beta ( A(t)+\eta Q(t) ) }{N(t)} ]C(t) , \\ \frac{dE}{dt}(t) = [ \frac{\beta ( A(t)+\eta Q(t) ) }{N(t)} ] [ S(t)+(1- \epsilon )C(t) ] -\gamma E(t) , \\ \frac{dA}{dt}(t) = q\gamma E(t)+r_{3}\sigma _{2}Q(t)-(\sigma _{1}+ \delta _{a})A(t), \\ \frac{dQ}{dt}(t) =(1-q)\gamma E(t)+r_{1}\sigma _{1}A(t)-(\sigma _{2}+ \delta _{q})Q(t), \\ \frac{dH}{dt}(t) =r_{2}\sigma _{1}A(t)+r_{4}\sigma _{2}Q(t)-(\sigma _{3}+ \delta _{h})H(t), \\ \frac{dR}{dt}(t) =(1-r_{1}-r_{2})\sigma _{1}A(t)+(1-r_{3}-r_{4}) \sigma _{2}Q(t)+\sigma _{3} H(t). \end{cases} $$

**Figure 1:**
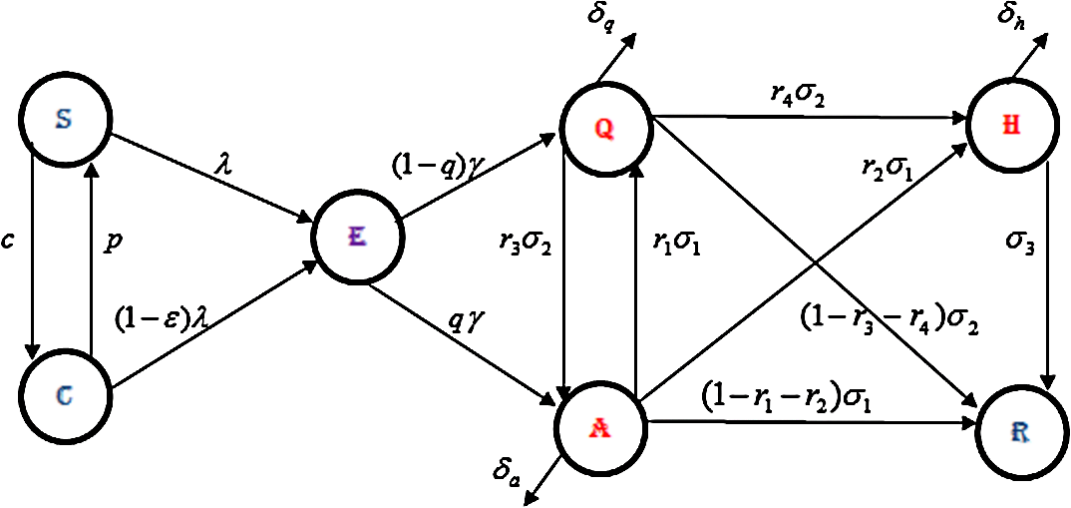
Model frame

The parameters are given in Table 1 and the model structure is provided in Fig. 1. A brief discussion on the model characteristics like boundedness and positivity of solutions is given in reference [36]. The authors in [36] calculated the greatest disease-free equilibrium point, which is defined by

$$ x_{0}= (S_{0},C_{0},0,0,0,0,0 )'= \biggl( \frac{pN_{0}}{p+c},\frac{cN_{0}}{p+c},0,0,0,0,0 \biggr)'. $$

Using the procedure given in [37], the matrices *F* and *V* for calculating the basic reproductive number are given by

2 $$\begin{array}{rl} & F=\left(\begin{array}{ccc} 0 & \beta \frac{S_{0}+(1-\epsilon )C_{0}}{N_{0}} & \eta \beta \frac{S_{0}+(1-\epsilon )C_{0}}{N_{0}}\\ 0 & 0 & 0\\ 0 & 0 & 0 \end{array}\right),\\ & V=\left(\begin{array}{ccc} \gamma & 0 & 0\\ -q\gamma & \left(\sigma_{1}+\delta_{a}\right) & -r_{3}\sigma_{2}\\ -(1-q)\gamma & -r_{1}\sigma_{1} & \left(\sigma_{2}+\delta_{q}\right) \end{array}\right). \end{array}$$

As a consequence, they obtained the basic reproductive number as the spectral-radius of the generation matrix, $FV^{-1}$:

3 $$ \begin{aligned}[b] \mathcal{R}_{c}&=\rho \bigl(FV^{-1} \bigr) \\ & =\beta \frac{S_{0}+(1-\epsilon )C_{0}}{N_{0}} \frac{ (r_{1}\sigma _{1}\eta +(\sigma _{2}+\delta _{q}) )q+ ((\sigma _{1}+\delta _{a})\eta +\sigma _{2}r_{3} )(1-q)}{(\sigma _{1}+\delta _{a})(\sigma _{2}+\delta _{q})-r_{1}r_{3}\sigma _{1}\sigma _{2}}. \end{aligned} $$

Here $\rho (\cdot )$ denotes the spectral-radius operator.

**Table 1:** Model parameters and their description

Parameter	Description
*β*	Contact rate
*η*	Relative transmissibility of quarantined infected carrier
*p*	Rate of transition from C(t) to S(t)
*c*	Confinement rate
*ϵ*	Confinement efficacy
*γ*	Rate of transition from E(t) to Q(t)
*q*	Rate of exposed individuals becoming quarantined
$\sigma _{1}$	Rate of transition from A(t) to Q(t)
$\sigma _{2}$	Transition rate from Q(t) to A(t)
$\sigma _{3}$	Rate of transition from hospitalized to recovered group
$r_{1}$	Fraction of A(t) becoming quarantined humans
$r_{2}$	Rate of unquarantined infected humans going to be hospitalized
$r_{3}$	Rate of quarantined infected humans moving to unquarantined infected humans
$r_{4}$	Rate of quarantined infected humans going to be hospitalized
$\delta _{a}$	Rate of 2019-nCOV deaths in unquarantined infected humans
$\delta _{q}$	Rate of 2019-nCOV deaths in quarantined infected humans
$\delta _{h}$	Rate of 2019-nCOV deaths in hospitalized

### Lemma 1

*If* $\mathcal{R}_{c}<1$, *the epidemic*-*free equilibrium* $x_{0}$ *is locally asymptotically stable and unstable if* $\mathcal{R}_{c}>1$.

### Proof

First, we observe that the last two equations of (1) are not coupled to the rest of equations of the model. As the total population size, $N_{0}$, is constant, we get $S+C=N_{0}-(E+A+Q+H+R)$. Then the local stability of the given system (1) can be analyzed via remaining model of variables (*E*, *A*, *Q*). As a consequence, we establish that the Jacobian matrix related to these variables is written by

$$J=\left(\begin{array}{ccc} -\gamma & \beta \frac{\left(S_{0}+(1-\epsilon )C_{0}\right)}{N_{0}} & \beta \eta \frac{\left(S_{0}+(1-\epsilon )C_{0}\right)}{N_{0}}\\ q\gamma & -(\sigma_{1}+\delta_{a}) & r_{3}\sigma_{2}\\ (1-q)\gamma & r_{1}\sigma_{1} & -(\sigma_{2}+\delta_{q}) \end{array}\right).$$

The roots of the next characteristic polynomial correspond to the eigenvalues of $\mathcal{J}$:

$$ P_{\mathcal{J}}(x)=x^{3}+a_{2}x^{2}+a_{1}x+a_{0}, $$

where

$$\begin{aligned}& a_{2}=\gamma +k_{2}+k_{1}, \\& a_{2}=(k_{1}k_{2}-\sigma _{1} \sigma _{2}r_{1}r_{3}) \\& \hphantom{a_{2}=}{}\times \biggl[ \frac{N_{0}k_{2}+N_{0}k_{1}}{N_{0}(k_{1}k_{2}-\sigma _{1}\sigma _{2}r_{1}r_{3})} \gamma +1-\gamma \frac{ (\eta (1-q)+q )}{q(k_{2}+r_{1}\sigma _{1}\eta )+(1-q)(\eta k_{1}+r_{3}\sigma _{2})} \mathcal{R}_{c} \biggr] \end{aligned}$$

and $a_{0}=\gamma (k_{1}k_{2}-r_{1}r_{3}\sigma _{1}\sigma _{2} ) (1-R_{c} )$. Note that $a_{2}$ is always positive. $a_{0}$ and $a_{1}$ are positive as long as $\mathcal{R}_{c}<1$. Therefore, all eigenvalues of $\mathcal{J}$ have negative real parts. Consequently, the epidemic-free equilibrium $x_{0}$, is locally asymptotically stable if $\mathcal{R}_{c}<1$. □

Excluding confinement measures, i.e. if $\epsilon =0$, $\mathcal{R}_{c}$ is convergent to the basic reproductive number, $\mathcal{R}_{0}$, written by

4 $$ \begin{aligned} \mathcal{R}_{0}=\beta \frac{ (r_{1}\sigma _{1}\eta +(\sigma _{2}+\delta _{q}) )q+ ((\sigma _{1}+\delta _{a})\eta +\sigma _{2}r_{3} )(1-q)}{(\sigma _{1}+\delta _{a})(\sigma _{2}+\delta _{q})-r_{1}r_{3}\sigma _{1}\sigma _{2}}. \end{aligned} $$

Using (3), it follows that

5 $$ \mathcal{R}_{c}=\mathcal{R}_{0} \biggl( \frac{S_{0}+(1-\epsilon )C_{0}}{N_{0}} \biggr)=\mathcal{R}_{0} \biggl( \frac{p+(1-\epsilon )c}{p+c} \biggr). $$

## Model calibration and forecasting

**Figure 2:**
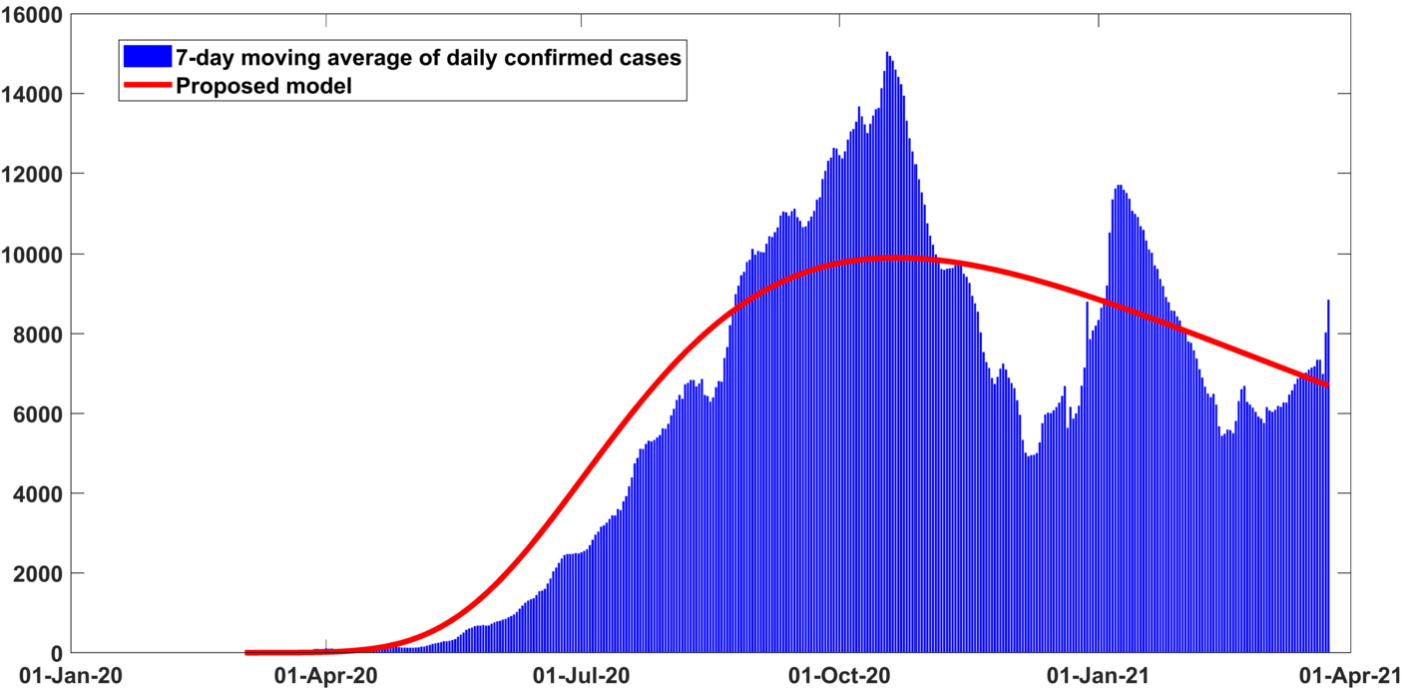
Output of the model performance fitting for daily cases of infection in Argentina from March 03, 2020 to March 29, 2021

A recently proposed optimization scheme, which is an evolution of Levenberg–Marquardt one [36, 38] and depends on trust-region-reflective (TRR) scheme, is employed to perform the model (1) calibration. This robust optimization method can be applied for simulating non-linear least-squares problems. The implementation of the scheme is done with the help of *lsqcurvefit* function, which is included in Optimization Toolbox from MATLAB. Possible parameter values are calculated by this function. The real data of daily collected cases in Argentina are collected from the trusted data website which can be verified in [39]. We used 7-day running average of the given reported COVID-19 cases analyzed in the calibration of model because of changeable structure of real data as can be seen in Fig. 2. The daily testing in Argentina is really inconsistent as in all nations. By the early updates, the total population size of Argentina is around 45,481,402 [39]. As initial population amount, we get $S(0)=45{,}481{,}396$, $C(0) = 0$, $E(0) = 0$, $A(0) = 0$, $H(0) = 0$, $R(0) = 0$ and $Q(0) = 1$. The numerical solutions are achieved solving the following optimization equation:

6 $$ \min_{\psi }\big\| \bigl(NPC_{predict}(t),NRC_{predict}(t) \bigr)- (NPC_{data},NRC_{data} )\big\| , $$

where $\psi = \lbrace \beta ,\sigma _{1},\sigma _{2},\sigma _{3},r_{1},r_{2},r_{3},r_{4}, \delta _{a},\delta _{q},\delta _{h},\eta ,p,c,\epsilon ,\gamma ,q \rbrace $ is shown in Table 2.

**Table 2:** Model parameters calibration by using the mentioned scheme

Parameters	Probable range	Base value	TRR output	Reference
*β*	0.5–1.5	0.5	1.2757	Fitted
$\sigma _{1}$	0.001–0.1	0.03	0.01	Fitted
$\sigma _{2}$	0.1–0.9	0.3	0.3488	Fitted
$\sigma _{3}$	0.1–0.9	0.3	0.6917	Fitted
$r_{1}$	0.1–0.9	0.5	0.302	Estimated
$r_{2}$	0.1–0.9	0.5	0.302	Estimated
$r_{3}$	0.1–0.9	0.5	0.2227	Estimated
$r_{4}$	0.1–0.9	0.5	0.3172	Estimated
$\delta _{a}$	0.001–0.1	0.01	0.1	Fitted
$\delta _{q}$	0.001–0.1	0.01	0.09	Fitted
$\delta _{h}$	0.001–0.1	0.01	0.0998	Fitted
*p*	0.0005–0.1	0.05	0.00051	Estimated
*η*	0.4–0.6	0.5	0.5077	Fitted
*c*	0.001–0.1	0.005	0.0155	Estimated
*γ*	1/14–1/3	1/5.1	0.1673	Fitted
*ϵ*	0.1–0.99	0.7	0.8417	Estimated
*q*	0.001–0.5	0.2	0.1181	Estimated

**Figure 3:**
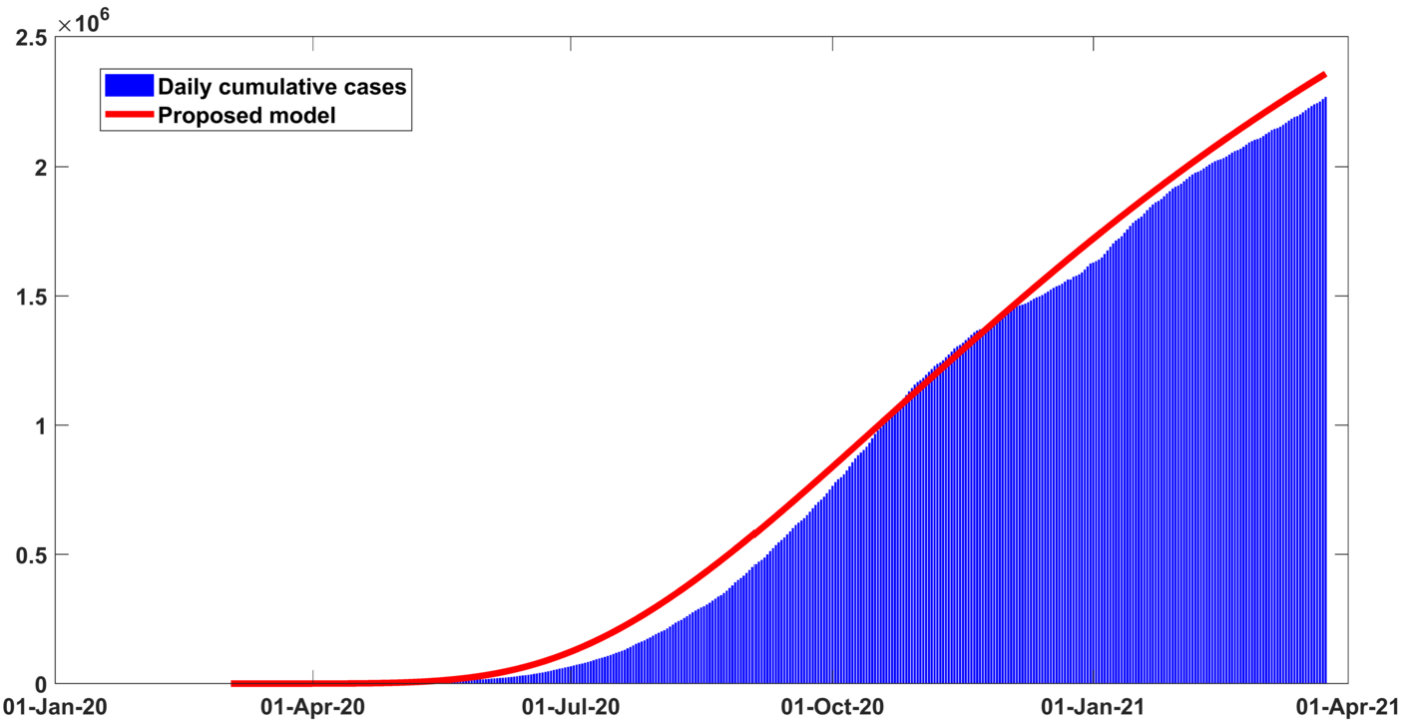
Output of the model performance fitting for cumulative cases of infection in Argentina from March 03, 2020 to March 29, 2021

**Figure 4:**
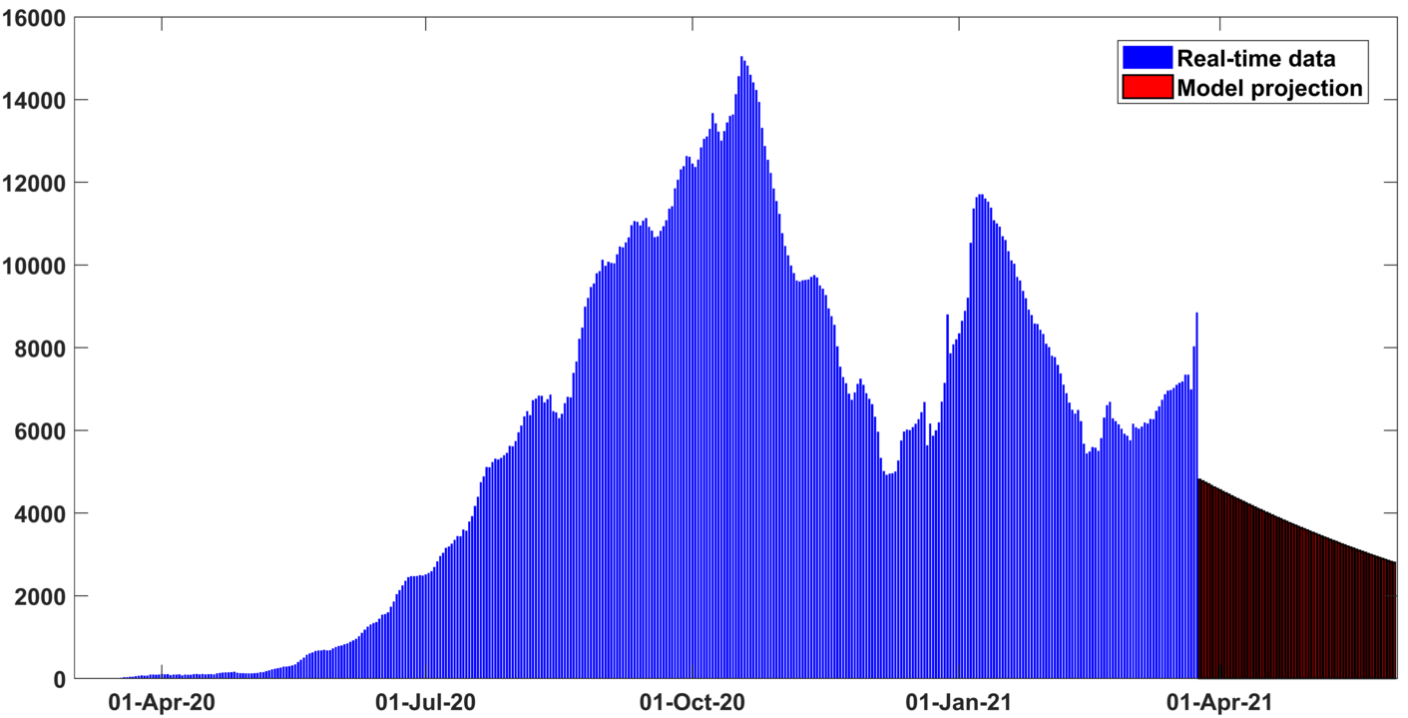
New daily reported cases projected and calibrated for Argentina from early March 2020 to late May 2021

**Figure 5:**
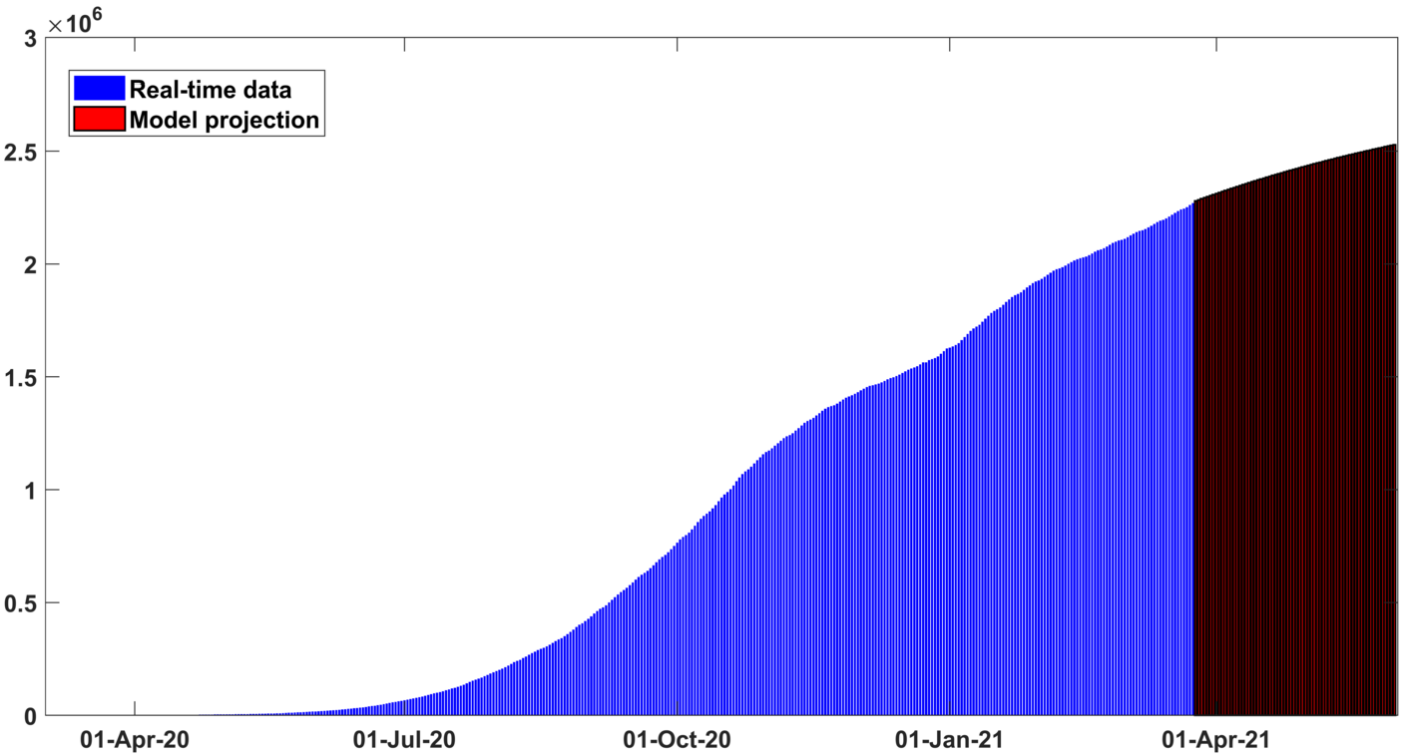
Cumulative infected cases fitted and projected for Argentina from early March to 2020 late May 2021

In Figs. 2 and 3 it is shown that our model fits really well to the given real data of Argentina. The estimated value of the basic reproductive number $\mathcal{R}_{0}$ is about ∼1.41 (95% CI: 1.2–1.6) as of March 10, 2021. This value could change (increase or decrease) in future cause of the new wave of Covid-19 which will totally depend on our health care measures. The disease could fade out at the end of December 2021. All necessary parameter values aspects applied to justify this scenario are mentioned in Table 2. In Fig. 4 the new daily and reported cases projected in Argentina from March 2020 to late May 2021 are given. In Fig. 5 cumulative infected cases and projected in the same dates are provided.

## Atangana–Baleanu fractional-order model

From the above integer-order simulations, we can see that the given model is working good to project real data for future. However, the integer-order model is not giving us much varieties in our predictions. In such cases, fractional-order derivatives always provide us a chance to obtain better predictions under the given real-data range. Motivated by this fact, we replace the above classical model into Atangana–Baleanu type fractional-order model which is defined under the Mittag-Leffler kernel.

### Preliminaries

Some important definitions are recalled here.

#### Definition 1

([40])

Given $\mathcal{S}\in \mathcal{H}^{1} (u, v )$, where $v>u$ and $0\leq \varPhi \leq 1$, the non-classical type Atangana–Baleanu (AB) derivative is stated as

7 $$ { ^{ABC}_{u}D^{\varPhi }_{t}} \bigl(\mathcal{S} (t ) \bigr)=\frac{\mathrm{ABC} [\varPhi ]}{1-\varPhi } \int ^{t}_{u}{ \mathcal{S}^{\prime } (\eta )E_{\varPhi } \biggl[\varPhi \frac{{ (t-\eta )}^{\varPhi }}{\varPhi -1} \biggr]\,d\eta }, $$

where $\mathrm{ABC} [\varPhi ]$ verifying $\mathrm{ABC} [0 ] = \mathrm{ABC} [1 ]= 1$ designates the normalization function and $E_{\varPhi }(\cdot)$ is the Mittag-Leffler function with one-parameter.

#### Definition 2

([40])

The non-classical type AB integral for normalization function $\mathrm{ABC} [\varPhi ]$ is set as

8 $$ ^{ABC}_{u}I^{\varPhi }_{t} \bigl( \mathcal{S} (t ) \bigr)= \frac{1-\varPhi }{\mathrm{ABC} [\varPhi ]}\mathcal{S} (t )+ \frac{\varPhi }{ \Gamma (\varPhi )\mathrm{ABC} [\varPhi ] } \int ^{t}_{u}{\mathcal{S} (\eta ){ (t-\eta )}^{ \varPhi -1}\,d\eta }. $$

#### Lemma 2

([40])

*The solution of the given system for* $0<\varPhi <1$

$$\begin{aligned}& {}^{\mathcal{ABC}}\mathbf{D}_{0}^{\varPhi }x(t) = z(t),\quad t\in [0, T], \\& x(0) = x_{0}, \end{aligned}$$

*is stated as*

$$ x(t)=x_{0}+\frac{(1-\varPhi )}{\mathrm{ABC}(\varPhi )}z(t)+ \frac{\varPhi }{\mathrm{ABC}(\varPhi )\Gamma (r)} \int _{0}^{t} (t- \omega )^{\varPhi -1}z( \omega )\,d\omega . $$

#### Lemma 3

([41])

*If* $0 < \varPhi < 1$ *and* $a_{1}$ *is a non*-*negative integer*, *then there exist constants* $C_{\varPhi , 1}>0$ *and* $C_{\varPhi , 2}>0$ *only dependent on* *Φ*, *such that*

$$ (a_{1} + 1)^{\varPhi }- a_{1}^{\varPhi }\leq C_{\varPhi , 1}(a_{1} + 1)^{ \varPhi - 1} $$

*and*

$$ (a_{1} + 2)^{\varPhi + 1} - 2(a_{1} + 1)^{\varPhi + 1} + a_{1}^{ \varPhi + 1} \leq C_{\varPhi , 2}(a_{1} + 1)^{\varPhi - 1}. $$

#### Lemma 4

([41])

*Let us suppose* $\nu _{p, n} = (n - p )^{\varPhi - 1}$ ($p = 1, 2,\ldots , n - 1$) *and* $\nu _{p, n}= 0$ *for* $p \geq n$, $\varPhi , M, h, T> 0$, $a_{1}h\leq T$ *and* $a_{1}$ *is a positive integer*. *Let* $\sum_{p= a_{1}}^{p= n}\nu _{p, n}|e_{p}|= 0$ *for* $k> n \geq 1$. *If*

$$ \vert e_{n} \vert \leq Mh^{\varPhi }\sum _{p=1}^{n-1}\nu _{p,n} \vert e_{p} \vert + \vert \eta _{0} \vert ,\quad n= 1,2, \ldots,a_{1}, $$

*then*

$$ \vert e_{a_{1}} \vert \leq C \vert \eta _{0} \vert ,\quad a_{1}= 1,2,\ldots, $$

*where* *C* *is a positive constant independent of* $a_{1}$ *and* *h*.

### Unique solution existence for fractional-order model

Let consider the Banach space $\mathbf{Z}=\mathbf{X}\times \mathbf{X}\times \mathbf{X} \times \mathbf{X} \times \mathbf{X} \times \mathbf{X} \times \mathbf{X}$, where $\mathbf{X}=C[0, T]$ endowed with the norm-function $\|A\|=\|(S, C, E, A, Q, H, R)\|=\max_{t\in [0, T]}[|S(t)+|C(t)|+|E(t)|+|A(t)|+|Q(t)|+|H(t)|+|R(t)|]$.

#### Theorem 1

([42])

*Consider* **B** *to be a convex subset of* **Z** *and let* **F**, **G** *depict couple operators satisfying*

1. $\mathbf{F}u + \mathbf{G}u\in \mathbf{B}$ $\forall u\in \mathbf{B}$;
2. **F** *is a contraction*;
3. **G** *is compact and continuous*.

*Then* $\mathbf{F}u+\mathbf{G}u=u$ *possesses at least one solution*.*”*

Now the generalization of the classical model (1) in the Atangana–Baleanu fractional derivative sense reads as follows:

9 $$ \textstyle\begin{cases} {}^{ABC}D^{\varPhi }_{t} S(t) =pC(t)-cS(t)- [ \frac{\beta ( A(t)+\eta Q(t) ) }{N(t)} ]S(t), \\ {}^{ABC}D^{\varPhi }_{t}C(t) =cS(t)-pC(t)-(1-\epsilon ) [ \frac{\beta ( A(t)+\eta Q(t) ) }{N(t)} ]C(t) , \\ {}^{ABC}D^{\varPhi }_{t}E(t) = [ \frac{\beta ( A(t)+\eta Q(t) ) }{N(t)} ] [ S(t)+(1- \epsilon )C(t) ] -\gamma E(t) , \\ {}^{ABC}D^{\varPhi }_{t}A(t) = q\gamma E(t)+r_{3}\sigma _{2}Q(t)-( \sigma _{1}+\delta _{a})A(t), \\ {}^{ABC}D^{\varPhi }_{t}Q(t) =(1-q)\gamma E(t)+r_{1}\sigma _{1}A(t)-( \sigma _{2}+\delta _{q})Q(t), \\ {}^{ABC}D^{\varPhi }_{t}H(t) =r_{2}\sigma _{1}A(t)+r_{4}\sigma _{2}Q(t)-( \sigma _{3}+\delta _{h})H(t), \\ {}^{ABC}D^{\varPhi }_{t}R(t) =(1-r_{1}-r_{2})\sigma _{1}A(t)+(1-r_{3}-r_{4}) \sigma _{2}Q(t)+\sigma _{3} H(t), \end{cases} $$

where ${}^{ABC}D^{\varPhi }_{t}$ is the Atangana–Baleanu operator of fractional order *Φ*. Now we rewrite the right-hand side of model (9) as follows:

10 $$ \textstyle\begin{cases} \mathrm{f}_{1}(t,S, \ldots , R) =pC(t)-cS(t)- [ \frac{\beta ( A(t)+\eta Q(t) ) }{N(t)} ]S(t), \\ \mathrm{f}_{2}(t,S, \ldots , R) =cS(t)-pC(t)-(1-\epsilon ) [ \frac{\beta ( A(t)+\eta Q(t) ) }{N(t)} ]C(t) , \\ \mathrm{f}_{3}(t,S, \ldots , R) = [ \frac{\beta ( A(t)+\eta Q(t) ) }{N(t)} ] [ S(t)+(1- \epsilon )C(t) ] -\gamma E(t) , \\ \mathrm{f}_{4}(t,S, \ldots , R) = q\gamma E(t)+r_{3}\sigma _{2}Q(t)-( \sigma _{1}+\delta _{a})A(t), \\ \mathrm{f}_{5}(t,S, \ldots , R) =(1-q)\gamma E(t)+r_{1}\sigma _{1}A(t)-( \sigma _{2}+\delta _{q})Q(t), \\ \mathrm{f}_{6}(t,S, \ldots , R) =r_{2}\sigma _{1}A(t)+r_{4}\sigma _{2}Q(t)-( \sigma _{3}+\delta _{h})H(t), \\ \mathrm{f}_{7}(t,S, \ldots , R) =(1-r_{1}-r_{2})\sigma _{1}A(t)+(1-r_{3}-r_{4}) \sigma _{2}Q(t)+\sigma _{3} H(t). \end{cases} $$

By using (10), we have

11 $$\begin{aligned}& {}^{\mathcal{ABC}}\mathbf{D}_{t}^{\varPhi } \mathcal{A}(t) = \Phi \bigl(t, \mathcal{A}(t) \bigr), \quad t\in [0, \tau ], 0< \varPhi \leq 1, \\& \mathcal{A}(0) = \mathcal{A}_{0}. \end{aligned}$$

According to Lemma 2, (11) yields

12 $$\begin{aligned} \mathcal{A}(t) =&\mathcal{A}_{0}(t)+ \bigl[\Phi \bigl(t, \mathcal{A}(t) \bigr)- \Phi _{0}(t) \bigr] \frac{(1-\varPhi )}{\mathrm{ABC}(\varPhi )} \\ &{}+ \frac{\varPhi }{\Gamma (\varPhi ) \mathrm{ABC}(\varPhi )} \int _{0}^{t} (t- \omega )^{\varPhi -1} \Phi \bigl(\omega , \mathcal{A}(\omega ) \bigr)\,d\omega , \end{aligned}$$

where

13 $$\begin{aligned}& \begin{aligned} &\mathcal{A}(t)= \textstyle\begin{cases} S(t), \\ C(t), \\ E(t), \\ A(t), \\ Q(t), \\ H(t), \\ R(t) ,\end{cases}\displaystyle \qquad \mathcal{A}_{0}(t)= \textstyle\begin{cases} S_{0}, \\ C_{0}, \\ E_{0}, \\ A_{0}, \\ Q_{0}, \\ H_{0}, \\ R_{0}, \end{cases}\displaystyle \qquad \Phi \bigl(t, \mathcal{A}(t) \bigr)= \textstyle\begin{cases} \mathrm{f}_{1}(t,S, \ldots , R), \\ \mathrm{f}_{2}(t,S, \ldots , R), \\ \mathrm{f}_{3}(t,S, \ldots , R), \\ \mathrm{f}_{4}(t,S, \ldots , R), \\ \mathrm{f}_{5}(t,S, \ldots , R), \\ \mathrm{f}_{6}(t,S, \ldots , R), \\ \mathrm{f}_{7}(t,S, \ldots , R), \end{cases}\displaystyle \\ &\Phi _{0}(t)= \textstyle\begin{cases} \mathrm{f}_{1}(0,S_{0}, \ldots, R_{0}), \\ \mathrm{f}_{2}(0,S_{0}, \ldots, R_{0}), \\ \mathrm{f}_{3}(0,S_{0}, \ldots, R_{0}), \\ \mathrm{f}_{4}(0,S_{0}, \ldots, R_{0}), \\ \mathrm{f}_{5}(0,S_{0}, \ldots, R_{0}), \\ \mathrm{f}_{6}(0,S_{0}, \ldots, R_{0}), \\ \mathrm{f}_{7}(0,S_{0}, \ldots, R_{0}). \end{cases}\displaystyle \end{aligned} \end{aligned}$$

Applying (12) and (13), the two operators **F**, **G** are defined as

14 $$\begin{aligned}& \mathbf{F}(\mathcal{A}) = \mathcal{A}_{0}(t)+ \bigl[ \Phi \bigl(t, \mathcal{A}(t) \bigr)-\Phi _{0}(t) \bigr] \frac{(1-\varPhi )}{\mathrm{ABC}(\varPhi )}, \\& \mathbf{G}(\mathcal{A}) = \frac{\varPhi }{\Gamma (\varPhi ) \mathrm{ABC}(\varPhi )} \int _{0}^{t} (t- \omega )^{\theta -1}\Phi \bigl( \omega , \mathcal{A}(\omega ) \bigr)\,d\omega . \end{aligned}$$

We now state some basic axioms and the Lipschitzian hypothesis for showing the existence and uniqueness of a solution:

1. There are $C_{\Phi }, D_{\Phi }>0$ such that
  $$ \bigl\vert \Phi \bigl(t, \mathcal{A}(t) \bigr) \bigr\vert \leq C_{\Phi } \vert \mathcal{A} \vert +D_{\Phi }. $$
2. There is $L_{\Phi }>0$ such that $\forall \mathcal{A}, \bar{\mathcal{A}}\in \mathbf{Z}$ and it follows that
  $$ \bigl\vert \Phi (t, \mathcal{A})-\Phi (t, \bar{\mathcal{A}}) \bigr\vert \leq L_{\Phi } \bigl[ \vert \mathcal{A}-\bar{\mathcal{A}} \vert \bigr]. $$

#### Theorem 2

*Under the hypotheses* (H1), (H2), *Eq*. (12) *possesses at least one solution*, *which implies that fractional model* (9) *possesses an equal number of solutions only if* $\frac{(1-\varPhi )}{\mathrm{ABC}(\varPhi )}L_{\Phi }<1$.

#### Proof

The proof is given in two parts.

*Step I*: Consider $\bar{\mathcal{A}}\in \mathbf{B}$, where $\mathbf{B}=\{\mathcal{A}\in \mathbf{Z}: \|\mathcal{A}\|\leq \rho , \rho >0\}$ is closed and convex. The operator **F** provided in (14) gives

15 $$\begin{aligned} \bigl\Vert \mathbf{F}(\mathcal{A})-\mathbf{F}( \bar{\mathcal{A}}) \bigr\Vert =& \frac{(1-\varPhi )}{\mathrm{ABC}(\varPhi )}\max_{t\in [0, \tau ]} \bigl\vert \Phi \bigl(t, \mathcal{A}(t) \bigr)-\Phi \bigl(t, \bar{\mathcal{A}}(t) \bigr) \bigr\vert , \\ \leq & \frac{(1-\varPhi )}{\mathrm{ABC}(\varPhi )}L_{\Phi } \Vert \mathcal{A}- \bar{\mathcal{A}} \Vert . \end{aligned}$$

Thus, **F** is a contraction.

*Step-II*: We want **G** to be relatively compact. Clearly, it is sufficient if **G** is equicontinuous and bounded. Indeed, **G** is continuous as Φ is continuous and for all $\mathcal{A}\in \mathbf{B}$, one has

16 $$\begin{aligned} \bigl\Vert \mathbf{G}(\mathcal{A}) \bigr\Vert =&\max _{t\in [0, \tau ]} \Vert \frac{\varPhi }{\Gamma (\varPhi )\mathrm{ABC}(\varPhi )} \int _{0}^{t} (t- \omega )^{\varPhi -1}\Phi \bigl( \omega , \mathcal{A}(\omega ) \bigr)\,d\omega \vert , \\ \leq & \frac{\varPhi }{\Gamma (\varPhi )\mathrm{ABC}(\varPhi )} \int _{0}^{ \tau }(\tau -\omega )^{\varPhi -1} \bigl\vert \Phi \bigl(\omega , \mathcal{A}(\omega ) \bigr) \bigr\vert d \omega , \\ \leq &\frac{\tau ^{\varPhi }}{\mathrm{ABC}(\varPhi )\Gamma (\varPhi )}[C_{ \Phi }\rho +D_{\Phi }]. \end{aligned}$$

Hence (16) shows the boundedness of **G**. For equicontinuity we assume $t_{1}>t_{2} \in [0, \tau ]$, so that

17 $$\begin{aligned}& \big|\mathbf{G}(\mathcal{A}(t_{1})-\mathbf{G}( \mathcal{A}(t_{1})\big| \\& \quad = \frac{\varPhi }{\mathrm{ABC}(\varPhi )\Gamma (\varPhi )} \biggl\vert \int _{0}^{t_{1}}(t_{1}-\omega )^{\varPhi -1}\Phi \bigl(\omega , \mathcal{A}(\omega ) \bigr)\,d\omega - \int _{0}^{t_{1}}(t_{1}-\omega )^{ \varPhi -1}\Phi \bigl(\omega , \mathcal{A}(\omega ) \bigr)\,d\omega \biggr\vert , \\& \quad \leq \frac{[C_{\Phi }\rho +D_{\Phi }]}{\mathrm{ABC}(\varPhi )\Gamma (\varPhi )} \bigl[t_{1}^{\varPhi }-t_{2}^{\varPhi } \bigr]. \end{aligned}$$

The right-hand side in (17) goes to zero as $t_{1}\rightarrow t_{2}$ and then

$$ \big|\mathbf{G}(\mathcal{A}(t_{1})-\mathbf{G}(\mathcal{A}(t_{1})\big| \rightarrow 0, \quad \text{as } t_{1}\rightarrow t_{2} $$

since **G** is continuous. Because of the boundedness and continuity of **G**, then **G** is uniformly continuous and bounded. According to the Arzelá–Ascoli theorem, **G** is thus relatively compact and therefore entirely continuous. Consequently, the integral equation (12) and also the method have at least one solution. □

We now proceed to show the uniqueness of the solution.

#### Theorem 3

*Assuming* $(H_{2})$, *Eq*. (12) *has a unique solution and this implies that model* (9) *has a unique solution only if* $[\frac{(1-\varPhi )L_{\Phi }}{\mathrm{ABC}(\varPhi )}+ \frac{\tau ^{\varPhi }L_{\Phi }}{\mathrm{ABC}(\varPhi )\Gamma (\varPhi )} ]<1$.

#### Proof

Consider $\mathbf{T}:\mathbf{Z}\rightarrow \mathbf{Z}$ defined as

18 $$\begin{aligned} \mathbf{T}\mathcal{A}(t) =&\mathcal{A}_{0}(t)+ \bigl[ \Phi \bigl(t, \mathcal{A}(t) \bigr)-\Phi _{0}(t) \bigr] \frac{(1-\varPhi )}{\mathrm{ABC}(\varPhi )} \\ &{}+ \frac{\varPhi }{\mathrm{ABC}(\varPhi )\Gamma (\varPhi )} \int _{0}^{t} (t- \omega )^{\varPhi -1} \Phi \bigl(\omega , \mathcal{A}(\omega ) \bigr)\,d\omega ,\quad t \in [0, \tau ]. \end{aligned}$$

Given $\mathcal{A}, \bar{\mathcal{A}} \in \mathbf{Z}$, then one can take

19 $$\begin{aligned} \Vert \mathbf{T}\mathcal{A}-\mathbf{T}\bar{\mathcal{A}} \Vert \leq &\frac{(1-\varPhi )}{\mathrm{ABC}(\varPhi )} \max_{t\in [0, \tau ]} \bigl\vert \Phi \bigl(t, \mathcal{A}(t) \bigr)-\Phi \bigl(t, \bar{\mathcal{A}}(t) \bigr) \bigr\vert , \\ &{}+\frac{\varPhi }{\Gamma (\varPhi )\mathrm{ABC}(\varPhi )} \\ &{}\times \max_{t \in [0, \tau ]} \biggl\vert \int _{0}^{t} (t-\omega )^{\varPhi -1} \Phi \bigl( \omega , \mathcal{A}(\omega ) \bigr)\,d\omega - \int _{0}^{t} (t-\omega )^{ \varPhi -1} \Phi \bigl( \omega , \bar{\mathcal{A}}(\omega ) \bigr)\,d\omega \biggr\vert , \\ \leq &\Xi \Vert \mathcal{A}-\bar{\mathcal{A}} \Vert , \end{aligned}$$

where

20 $$\begin{aligned} \Xi = \biggl[\frac{(1-\varPhi )L_{\Phi }}{\mathrm{ABC}(\varPhi )}+ \frac{\tau ^{\varPhi }L_{\Phi }}{\Gamma (\varPhi )\mathrm{ABC}(\varPhi )} \biggr]. \end{aligned}$$

Thus, **T** is a contraction. Therefore, Eq. (12) possesses a unique solution and so does model (9). □

### Derivation of solution

To date, number of numerical approximation algorithms have been proposed to solve the various kind of models describing the real world problems. When we apply such algorithms the analysis of convergence and stability are two important aspects of the effectiveness of the method. For solving our model, we are going to use predictor–corrector method, which has been defined probably for all fractional-order operators. Here we implement this algorithm in the sense of Atangana–Baleanu operator for writing the solution of the proposed COVID-19 model. For more information about this technique see Ref. [43]. We first recall Eq. (11) and consider

21 $$\begin{aligned}& {}^{\mathcal{ABC}}\mathbf{D}_{t}^{\varPhi } \mathcal{A}(t) = \Phi \bigl(t, \mathcal{A}(t) \bigr), \quad t\in [0, \tau ], 0< \varPhi \leq 1, \\& \mathcal{A}(0) = \mathcal{A}_{0}. \end{aligned}$$

The fractional Volterra integral equation is stated as

22 $$ \mathcal{A}_{i+1}= \mathcal{A}_{0}+ (1- \varPhi )\Phi (t_{i+1}, \mathcal{A}_{i+1})+ \frac{\varPhi }{\Gamma (\varPhi )} \int _{0}^{t_{i+1}}(t_{i+1}-s)^{ \varPhi - 1} \Phi \bigl(s, \mathcal{A}(s) \bigr)\,ds. $$

According to the method proposed in [43] for $\varPhi \in [0, 1]$, $0\leq t\leq T$ and considering $h= T/N$ and $t_{n}= nh$, for $n= 0, 1, 2,\ldots,N\in \mathbb{Z}^{+}$, the corrector formula for the given system is

23 $$ \mathcal{A}_{i+1}= \mathcal{A}_{0}+ \frac{\varPhi h^{\zeta }}{\Gamma (\varPhi + 2)} \Biggl(a_{i+1,i+1}\Phi \bigl(t_{i+1}, \mathcal{A}_{i+1}^{P} \bigr)+ \sum _{j=0}^{i}a_{i+1,j}\Phi (t_{j}, \mathcal{A}_{j}) \Biggr), $$

where

24 $$\begin{aligned} a_{i+1, j}= \textstyle\begin{cases} i^{\varPhi +1}- (i- \varPhi ){(i+ 1)}^{\varPhi } \quad \text{if } j=0, \\ {(i- j+ 2)}^{\varPhi + 1}+ {(i- j)}^{\varPhi +1}- 2{(i- j+1)}^{ \varPhi +1} \quad \text{if } 1\leq j\leq i, \\ 1, \quad j= i+1, \end{cases}\displaystyle \end{aligned}$$

and

$$ a_{i+1, i+1}= 1+ \frac{(1-\varPhi )\Gamma (\varPhi +2)}{\varPhi h^{\varPhi }}. $$

The predictor formula is attained as

25 $$ \mathcal{A}_{i+1}^{P}= \mathcal{A}_{0}+ \frac{ h^{\varPhi }}{\Gamma (\varPhi )} \sum_{j=0}^{i}b_{i+1,j} \Phi (t_{j}, \mathcal{A}_{j}), $$

where

26 $$\begin{aligned} b_{i+1, j}= \textstyle\begin{cases} -(i- j)^{\varPhi }+ (i- j+ 1)^{\varPhi }, \quad j=0,\ldots,i-1, \\ 1+ \frac{(1- \varPhi )\Gamma (\varPhi )}{h^{\varPhi }}, \quad j=i. \end{cases}\displaystyle \end{aligned}$$

Hence the corrector formulas for the given model (9) are stated as

27 $$\begin{aligned}& S_{i+1} = S_{0}+ \frac{\varPhi h^{\varPhi }}{\Gamma (\varPhi + 2)} \Biggl((a_{i+1,i+1})f_{1} \bigl(t_{i+1}, S_{i+1}^{P}, C_{i+1}^{P}, E_{i+1}^{P}, A_{i+1}^{P}, Q_{i+1}^{P}, H_{i+1}^{P}, R_{i+1}^{P} \bigr) \\& \hphantom{S_{i+1} =}{} + \sum_{j=0}^{i}(a_{i+1,j})f_{1}(t_{i}, S_{j}, C_{j}, E_{j}, A_{j}, Q_{j}, H_{j}, R_{j}) \Biggr), \\& C_{i+1} = C_{0}+ \frac{\varPhi h^{\varPhi }}{\Gamma (\varPhi + 2)} \Biggl((a_{i+1,i+1})f_{2} \bigl(t_{i+1}, S_{i+1}^{P}, C_{i+1}^{P}, E_{i+1}^{P}, A_{i+1}^{P}, Q_{i+1}^{P}, H_{i+1}^{P}, R_{i+1}^{P} \bigr) \\& \hphantom{C_{i+1} =}{} + \sum_{j=0}^{i}(a_{i+1,j})f_{2}(t_{i}, S_{j}, C_{j}, E_{j}, A_{j}, Q_{j}, H_{j}, R_{j}) \Biggr), \\& E_{i+1} = E_{0}+ \frac{\varPhi h^{\varPhi }}{\Gamma (\varPhi + 2)} \Biggl((a_{i+1,i+1})f_{3} \bigl(t_{i+1}, S_{i+1}^{P}, C_{i+1}^{P}, E_{i+1}^{P}, A_{i+1}^{P}, Q_{i+1}^{P}, H_{i+1}^{P}, R_{i+1}^{P} \bigr) \\& \hphantom{E_{i+1} =}{} + \sum_{j=0}^{i}(a_{i+1,j})f_{3}(t_{i}, S_{j}, C_{j}, E_{j}, A_{j}, Q_{j}, H_{j}, R_{j}) \Biggr), \\& \begin{aligned}&A_{i+1} = A_{0}+ \frac{\varPhi h^{\varPhi }}{\Gamma (\varPhi + 2)} \Biggl((a_{i+1,i+1})f_{4} \bigl(t_{i+1}, S_{i+1}^{P}, C_{i+1}^{P}, E_{i+1}^{P}, A_{i+1}^{P}, Q_{i+1}^{P}, H_{i+1}^{P}, R_{i+1}^{P} \bigr) \\ &\hphantom{A_{i+1} =}{} + \sum_{j=0}^{i}(a_{i+1,j})f_{4}(t_{i}, S_{j}, C_{j}, E_{j}, A_{j}, Q_{j}, H_{j}, R_{j}) \Biggr), \end{aligned}\\& Q_{i+1} = Q_{0}+ \frac{\varPhi h^{\varPhi }}{\Gamma (\varPhi + 2)} \Biggl((a_{i+1,i+1})f_{5} \bigl(t_{i+1}, S_{i+1}^{P}, C_{i+1}^{P}, E_{i+1}^{P}, A_{i+1}^{P}, Q_{i+1}^{P}, H_{i+1}^{P}, R_{i+1}^{P} \bigr) \\& \hphantom{Q_{i+1} =}{} + \sum_{j=0}^{i}(a_{i+1,j})f_{5}(t_{i}, S_{j}, C_{j}, E_{j}, A_{j}, Q_{j}, H_{j}, R_{j}) \Biggr), \\& H_{i+1} = H_{0}+ \frac{\varPhi h^{\varPhi }}{\Gamma (\varPhi + 2)} \Biggl((a_{i+1,i+1})f_{6} \bigl(t_{i+1}, S_{i+1}^{P}, C_{i+1}^{P}, E_{i+1}^{P}, A_{i+1}^{P}, Q_{i+1}^{P}, H_{i+1}^{P}, R_{i+1}^{P} \bigr) \\& \hphantom{H_{i+1} =}{} + \sum_{j=0}^{i}(a_{i+1,j})f_{6}(t_{i}, S_{j}, C_{j}, E_{j}, A_{j}, Q_{j}, H_{j}, R_{j}) \Biggr), \\& R_{i+1} = R_{0}+ \frac{\varPhi h^{\varPhi }}{\Gamma (\varPhi + 2)} \Biggl((a_{i+1,i+1})f_{7} \bigl(t_{i+1}, S_{i+1}^{P}, C_{i+1}^{P}, E_{i+1}^{P}, A_{i+1}^{P}, Q_{i+1}^{P}, H_{i+1}^{P}, R_{i+1}^{P} \bigr) \\& \hphantom{R_{i+1} =}{} + \sum_{j=0}^{i}(a_{i+1,j})f_{7}(t_{i}, S_{j}, C_{j}, E_{j}, A_{j}, Q_{j}, H_{j}, R_{j}) \Biggr), \end{aligned}$$

where

28 $$ \begin{aligned} &S^{P}_{i+1} = S_{0}+ \frac{h^{\varPhi }}{\Gamma (\varPhi )}\sum_{j=0}^{i}b_{i+1, j}f_{1}(t_{j}, S_{j}, C_{j}, E_{j}, A_{j}, Q_{j}, H_{j}, R_{j}), \\ &C^{P}_{i+1} = C_{0}+ \frac{h^{\varPhi }}{\Gamma (\varPhi )} \sum_{j=0}^{i}b_{i+1, j}f_{2}(t_{j}, S_{j}, C_{j}, E_{j}, A_{j}, Q_{j}, H_{j}, R_{j}), \\ &E^{P}_{i+1} = E_{0}+ \frac{h^{\varPhi }}{\Gamma (\varPhi )} \sum_{j=0}^{i}b_{i+1, j}f_{3}(t_{j}, S_{j}, C_{j}, E_{j}, A_{j}, Q_{j}, H_{j}, R_{j}), \\ &A^{P}_{i+1} = A_{0}+ \frac{h^{\varPhi }}{\Gamma (\varPhi )} \sum_{j=0}^{i}b_{i+1, j}f_{4}(t_{j}, S_{j}, C_{j}, E_{j}, A_{j}, Q_{j}, H_{j}, R_{j}), \\ &Q^{P}_{i+1} = Q_{0}+ \frac{h^{\varPhi }}{\Gamma (\varPhi )} \sum_{j=0}^{i}b_{i+1, j}f_{5}(t_{j}, S_{j}, C_{j}, E_{j}, A_{j}, Q_{j}, H_{j}, R_{j}), \\ &H^{P}_{i+1} = H_{0}+ \frac{h^{\varPhi }}{\Gamma (\varPhi )} \sum_{j=0}^{i}b_{i+1, j}f_{6}(t_{j}, S_{j}, C_{j}, E_{j}, A_{j}, Q_{j}, H_{j}, R_{j}), \\ &R^{P}_{i+1} = R_{0}+ \frac{h^{\varPhi }}{\Gamma (\varPhi )} \sum_{j=0}^{i}b_{i+1, j}f_{7}(t_{j}, S_{j}, C_{j}, E_{j}, A_{j}, Q_{j}, H_{j}, R_{j}). \end{aligned} $$

#### Stability of the method

##### Theorem 4

*The given algorithm* (27)*–*(28) *is conditionally stable*.

##### Proof

Let $\tilde{\mathcal{A}_{0}}$, $\tilde{\mathcal{A}_{j}}$ ($j= 0,\ldots, i+1$) and $\tilde{\mathcal{A}}_{i+1}^{P}$ ($i= 0,\ldots,N-1$) be perturbations of $\mathcal{A}_{0}$, $\mathcal{A}_{j}$ and $\mathcal{A}_{i+1}^{P}$, respectively. Then the perturbation equations derived by using Eqs. (27) and (28) are given by

29 $$\begin{aligned}& \tilde{\mathcal{A}}^{P}_{i+1}= \tilde{ \mathcal{A}_{0}}+ \frac{h^{\varPhi }}{\Gamma (\varPhi )}\sum _{j=0}^{i}b_{i+1, j} \bigl( \Phi (t_{j}, \mathcal{A}_{j}+ \tilde{ \mathcal{A}_{j}})- \Phi (t_{j}, \mathcal{A}_{j}) \bigr), \end{aligned}$$

30 $$\begin{aligned}& \begin{aligned} &\tilde{\mathcal{A}}_{i+1}= \tilde{ \mathcal{A}_{0}}+ \frac{\varPhi h^{\varPhi }}{\Gamma (\varPhi + 2)} \Biggl(a_{i+1,i+1} \bigl( \Phi \bigl(t_{i+1}, \mathcal{A}_{i+1}^{P}+ \tilde{ \mathcal{A}}_{i+1}^{P} \bigr)- \Phi \bigl(t_{i+1}, \mathcal{A}_{i+1}^{P} \bigr) \bigr) \\ &\hphantom{\tilde{\mathcal{A}}_{i+1}=}{}+ \sum_{j=0}^{i}a_{i+1,j} \bigl( \Phi (t_{j}, \mathcal{A}_{j}+ \tilde{ \mathcal{A}}_{j})- \Phi (t_{j}, \mathcal{A}_{j}) \bigr) \Biggr). \end{aligned} \end{aligned}$$

According to the Lipschitz condition, we get

31 $$ \vert \tilde{\mathcal{A}}_{i+1} \vert \leq \varPhi _{0}+ \frac{ \varPhi h^{\varPhi }M}{\Gamma (\varPhi + 2)} \Biggl(a_{i+1, i+1} \bigl\vert \tilde{ \mathcal{A}}^{P}_{i+1} \bigr\vert +\sum _{j=1}^{i} a_{j, i+1} \vert \tilde{ \mathcal{A}}_{j} \vert \Biggr), $$

where $\varPhi _{0}= \max_{0\leq i\leq N}\{|\tilde{\mathcal{A}_{0}}|+ \frac{\varPhi h^{\varPhi }M a_{i,0}}{\Gamma (\varPhi + 2)}| \tilde{\mathcal{A}_{0}}|\}$. Also, from Eq. (3.18) in [41] we write

32 $$ \bigl\vert \tilde{\mathcal{A}}^{P}_{i+1} \bigr\vert \leq \eta _{0}+ \frac{ h^{\varPhi }M}{\Gamma (\varPhi )}\sum _{j=1}^{i}b_{j, i+1} \vert \tilde{ \mathcal{A}}_{j} \vert , $$

where $\eta _{0}= \max_{0\leq i\leq N}\{|\tilde{\mathcal{A}}_{0}|+ \frac{h^{\varPhi }M b_{n,0}}{\Gamma (\varPhi )}|\tilde{\mathcal{A}}_{0}| \}$. Substituting $|\tilde{\mathcal{A}}^{P}_{i+1}|$ from Eq. (32) into Eq. (31) we find

33 $$ \begin{aligned} \vert \tilde{\mathcal{A}}_{i+1} \vert &\leq \gamma _{0}+ \frac{\varPhi h^{\varPhi }M}{\Gamma (\varPhi + 2)}\sum _{j=1}^{i} \biggl( a_{i+1, j}+ \frac{h^{\varPhi }M a_{i+1, i+1}b_{i+1, j}}{\Gamma (\varPhi )} \biggr) \vert \tilde{\mathcal{A}}_{j} \vert \\ &\leq \gamma _{0}+ \frac{\varPhi h^{\varPhi }M C_{\varPhi , 2}}{\Gamma (\varPhi + 2)} \sum _{j=1}^{i}(i+ 1- j)^{\varPhi - 1} \vert \tilde{ \mathcal{A}_{j}} \vert , \end{aligned} $$

where $\gamma _{0}= \max \{\varPhi _{0}+ \frac{ \varPhi h^{\varPhi }M a_{i+1,i+1}}{\Gamma (\varPhi + 2)}\eta _{0} \}$. $C_{\varPhi ,2}>0$ is constant and only depends on *Φ* (see Lemma 3) and *h* is surmised to be small enough. From Lemma 4 we get $|\tilde{\mathcal{A}}_{i+1}|\leq C \gamma _{0}$, which finishes the requirement. □

### Graphical justifications

**Figure 6:**
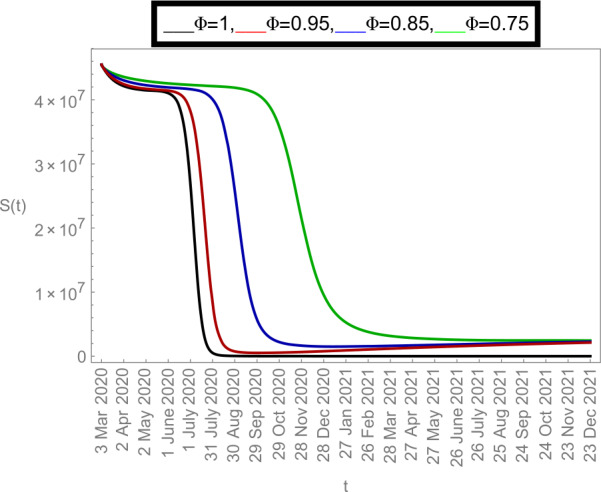
Dynamics of $S(t)$ population

**Figure 7:**
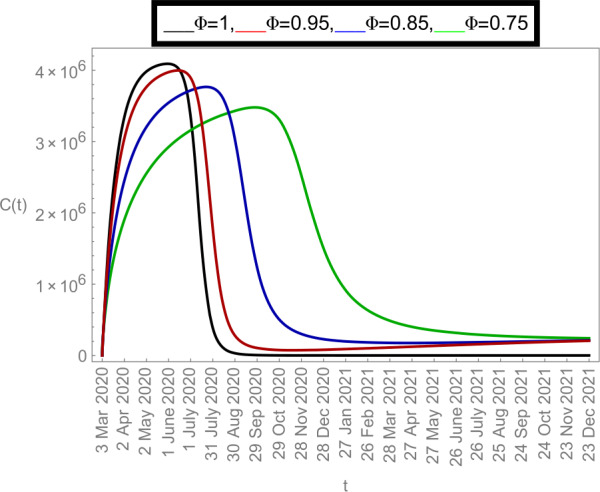
Dynamics of $C(t)$ population

**Figure 8:**
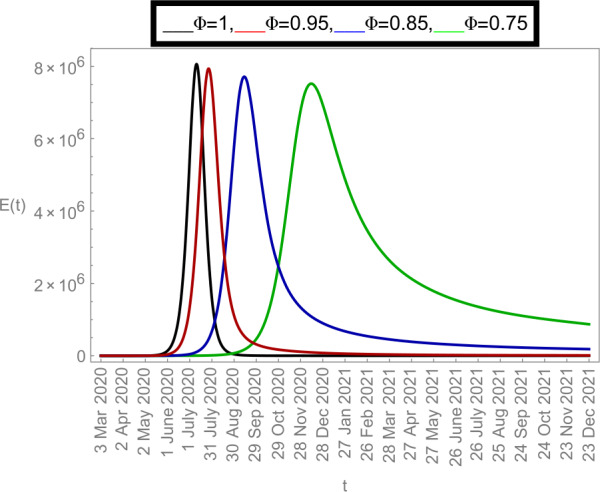
Dynamics of $E(t)$ population

**Figure 9:**
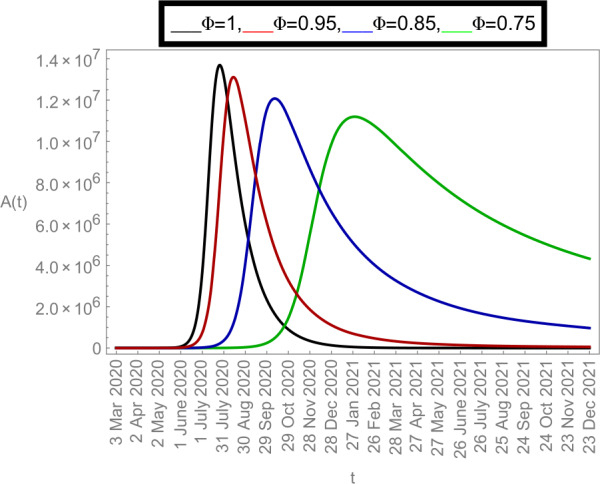
Dynamics of $A(t)$ population

**Figure 10:**
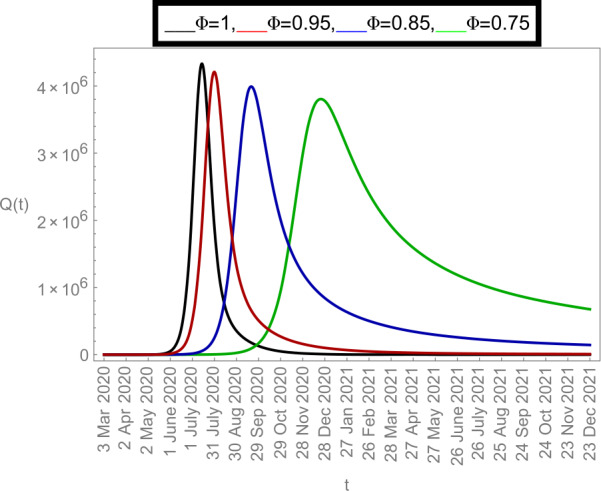
Dynamics of $Q(t)$ population

**Figure 11:**
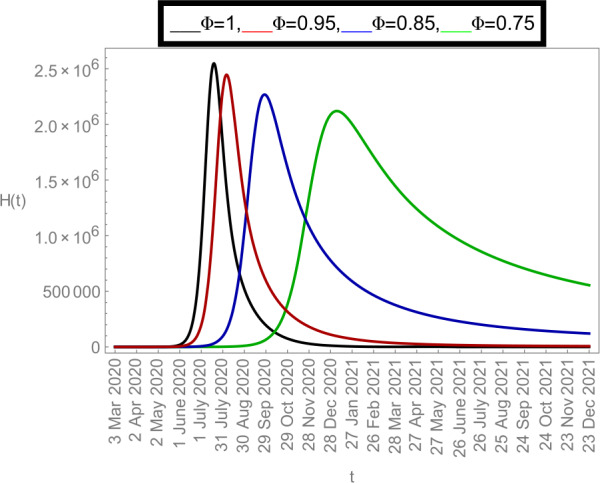
Dynamics of $H(t)$ population

**Figure 12:**
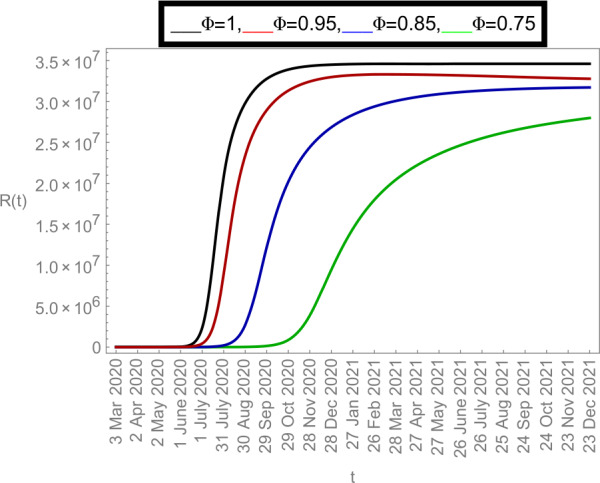
Dynamics of $R(t)$ population

Now we plot the fractional-order solution by considering the parameter values simulated in Table 2. This time we used *Mathematica* software. In Fig. 6, we plotted the susceptible population $S(t)$ and in Fig. 7, the plot of confined individuals $C(t)$ is given. We can observe that when time increases then the population of susceptible individuals is decreasing which is a good indication for Argentina. In Fig. 8, we plotted the nature of exposed individuals $E(t)$. We notice that when the fractional-order values decreases then the peak of $E(t)$ shifts to the next month. At fractional order $\varPhi =1$, a peak occurs at near around 1 *July* 2020 and when we shifted to lower values of fractional order then we receive the peak near around 31 *July* 2020, at $\varPhi =0.95$, near around 29 *Sep* 2020, at $\varPhi =0.85$, and near around 28 *Dec* 2020 when $\varPhi =0.75$. This shows how the fractional-order derivatives give us more varieties to predict the real-data structure for the future. Similarly, in Fig. 9, we plotted the nature of asymptomatic individuals $A(t)$ where we can see that at fractional-order $\varPhi =1$, peak occurs at near around 31 *July* 2020 and when we shift to lower values of fractional order then we receive the peak near around 15 *Aug* 2020 at $\varPhi =0.95$, near around 15 *Oct* 2020 at $\varPhi =0.85$, and near around 27 *Jan* 2021 when $\varPhi =0.75$. In this series, Fig. 10 plotted the nature of quarantined individuals $Q(t)$ where different peaks can be seen. Here at fractional order $\varPhi =1$, peak occurs at near around 15 *July* 2020 and when we shifted to lower values of fractional order then we obtain the peak around 31 *July* 2020 at $\varPhi =0.95$, around 29 *Sep* 2020 at $\varPhi =0.85$, and around 28 *Dec* 2020 at $\varPhi =0.75$. Figure 11 described the behavior of hospitalized individuals $H(t)$ for the given time range. Here at fractional order $\varPhi =1$, a peak occurs around 15 *July* 2020 and when we shifted to lower values of fractional order then we receive the peak around 31 *July* 2020 at $\varPhi =0.95$, around 29 *Sep* 2020 at $\varPhi =0.85$, and around 30 *Dec* 2020 at $\varPhi =0.75$. Finally, Fig. 12 shows the nature of recovered individuals $R(t)$ versus given time range.

All above given graphs confirm that the COVID-19 disease is under control in Argentina at this time stage (based on the given data simulations). However, it is well known that if the population does not follow all health care measures then the situation may change in the future. The given fractional-order model under Mittag-Leffler kernel gives very well outputs to predict the dynamics of COVID-19 in Argentina. We used different fractional-order values for the comparisons because an integer-order model can provide a single peak prediction which does not show the varieties in the predictions. Different fractional-order values give various predictions which may in the future be more accurate. This is the ultimate advantage of using fractional derivatives.

## Conclusion

In our observations, we have explored the dynamics of the most deathly disease of this decade, COVID-19 in Argentina by considering parameter values based on real data. Firstly, we considered the reported cases of this virus from *March* 03, 2020 to *March* 29, 2021 and projected it for the future time period by using our models. We proposed a Atangana–Baleanu type fractional-order model and solved it employing predictor–corrector (P-C) algorithm. After analyzing the biological nature of this virus, we formulated a mathematical structure to define its dynamics. We used a well-known effective optimization method based on the renowned trust-region-reflective (TRR) scheme to perform the model calibration. We plotted the real cases of COVID-19 and fitted our integer-order model with the data along with the calculation of basic reproductive number. In the simulations of fractional-order analysis, first we proved the existence of unique solution and then wrote the solution of the model along with the stability of the given P-C method. We performed separate graphs for every model class at different fractional-order values to predict the future dynamics of the virus in Argentina. Finally, we conclude that this virus is under control in Argentina for future under the given data range and all health care measures. In the future, the given estimated parameter values and models can be used to provide further predictions on the transmission of this virus. The proposed mathematical model is effective and trustable for future use. Not only from a mathematical point of view but also from a medical perspective, this research study may become very useful for the medical authorities to simulate the outbreaks of COVID-19 in Argentina for a future time period. Still, all the peak predictions which have been simulated in this paper, do not claim with 100% certainty that the dynamics of COVID-19 will be as it is. It may differ because of many health care measures (like isolation strategies, hospital facilities, and vaccine availability) which have been mentioned in this study.

## Data Availability

All the data is included in the paper.
